# Supplementary material for: Iranian livestock breeders’ knowledge, attitude, practice, and behavioral determinants related to brucellosis prevention
Source: PLOS Glob Public Health. 2025 Oct 8;5(10):e0004693. doi: 10.1371/journal.pgph.0004693 (PMC12507205; doi:10.1371/journal.pgph.0004693)
Supplement: S1 Checklist — (S1_Checklist.DOCX) [file pgph.0004693.s001.docx]

Inclusivity in global research

PLOS’ policy on inclusivity in global research aims to improve transparency in the reporting of research performed outside of researchers’ own country or community and ensures that PLOS publications reporting global research adhere to high standards for research ethics and authorship. Authors of relevant research articles may be asked to complete the questionnaire below, which outlines ethical, cultural, and scientific considerations specific to inclusivity in global research. This questionnaire may be requested when researchers have travelled to a different country to conduct research, if research uses samples collected in another country, research with Indigenous populations or their lands, or if research is on cultural artefacts. Researchers travelling to another country solely to use laboratory equipment will not normally be required to complete the questionnaire. However, the questionnaire can be requested at the journal’s discretion for any submission – if you have been requested to complete this questionnaire by the PLOS journal you submitted to, please do so.

Please complete the questionnaire below and include this as a Supporting Information file with your manuscript. Note that if your paper is accepted for publication, this checklist will be published with your article in the supporting information files. Please ensure that you reference the checklist in the main body of your manuscript. We suggest adding a subsection ‘Inclusivity in global research’ to your Methods section and adding the following sentence: “Additional information regarding the ethical, cultural, and scientific considerations specific to inclusivity in global research is included in the Supporting Information (SX Checklist)”

The questions have been designed to be applicable to a wide range of study types, and there are subsections for both human subjects research and non-human subjects research. If any of the questions are not relevant to your research please mark them as “N/A” as appropriate.

**Ethical considerations, permits and authorship**

*This section is applicable to all research types.*

Provide details as to who granted permissions and/or consent for the study to take place in the Methods section of your manuscript. This should include the names of **all** ethics boards, governmental organizations, community leaders or other bodies that provided approval for the study. If individuals provided approval refer to these people by their role or title but do not list their name(s).

Reported on page number: Page 7 line 134

The faculty of medical sciences' ethical committee board at Tarbiat Modares University approved the present research (IRB number: IR.MODARES.REC.1397.001).The procedure was performed according to the relevant guidelines, regulations, ethical standards of the responsible committee, which approved the study at Tarbiat Modares University, the Iranian Registry of Clinical Trials, and the Declaration of Helsinki (the revised version in 2000). The researchers confirm that all methods were performed following the relevant guidelines and regulations.

If there were any deviations from the study protocol after approval was obtained please provide details of these changes in the Methods section of your manuscript.
Did this study involve local collaborators that are residents of the country where the research was conducted or members of the community studied? If you do not have any authors from said communities, please provide an explanation for this below.

There were no deviations from the approved study protocol. All procedures were conducted in accordance with the original protocol approved by the relevant ethics committee.

Reported on page number:

Everyone listed as an author should meet PLOS’ criteria for authorship and all individuals who meet the criteria should be included in the author byline, rather than the acknowledgements. For further information please see the journal’s Authorship Policy.

**Human subjects research (e.g. health research, medical research, cross-cultural psychology)**

Did you obtain written informed consent from a representative of the local community or region before the research took place? How did you establish who speaks for the community? Details of written informed consent obtained from study participants should be reported separately in the Methods section of your manuscript.

Participants were informed about the present study's purpose before the investigation. Volunteer animal breeders participated in this study. The main researcher read the form for illiterate breeders; then breeders stamped the consent form. The research data are kept confidential. All participants had a unique code and could withdraw from the study at any time. Participants provided written informed consent and were ensured that their responses would remain confidential.

How did members of the local community provide input on the aims of the research investigation, its methodology, and its anticipated outcome(s)?

When engaging with the local community, how did you ensure that the informed consent documents and other materials could be understood by local stakeholders?

To ensure comprehension among local stakeholders, all informed consent documents and related materials were prepared in Persian, the native language of the participants. The content was written in clear, accessible language, avoiding technical jargon to facilitate understanding. Additionally, participants were given the opportunity to ask questions, and clarifications were provided verbally when needed. This approach helped guarantee that individuals were fully informed about the study’s purpose, procedures, and their rights before providing consent.

Members of the local community contributed to the research by providing cultural and contextual insights throughout the development of the study. Since the research was conducted in Iran and the research team consisted of Iranian scholars familiar with the local context, community perspectives were inherently integrated into the formulation of the study aims, the selection of appropriate methodologies, and the interpretation of anticipated outcomes. Informal consultations with local professionals and stakeholders also helped ensure that the study design was culturally sensitive and relevant to the target population.

Will the findings of the research be made available in an understandable format to stakeholders in the community where the study was conducted (e.g. via a presentation, summary report, copies of publications, etc.)? Please provide details of how this will be achieved.

Yes, the findings of the research will be shared with stakeholders in the community in a format that is accessible and easy to understand. A summary report in Persian will be prepared, highlighting the key results and implications of the study in non-technical language. This report will be distributed to local community organizations, schools, and relevant stakeholders. Additionally, we plan to present the findings in a community meeting or seminar to allow for discussion and feedback. Copies of academic publications resulting from the research will also be made available upon request.

**Non-human subjects research using specimens/ animals collected as part of the study, or those housed in archival collections. Examples include archaeology, paleontology, botany and zoology.**

Did the permission you obtained from a local authority to perform the study include an agreement on access to outputs and benefit sharing? This may include procedures to enable fair distribution of the benefits and resources arising from the research performed. Please include any details of Prior Informed Consent and Benefit Sharing Agreements obtained. These may be required by field-specific regulations, for example the Convention on Biological Diversity (CBD) and the associated Nagoya Protocol.

Not applicable.

If the material used in your study was imported, please A) provide the year it was imported and B) indicate whether permits were obtained to import/export the materials used, C) provide details of any permits obtained. If this information is not available, please indicate this.

Not applicable.

If you used archival specimens, please state how the material used in your study was acquired by the institute it is held in and provide details of any permits obtained for the original excavations/ sample collection. If this information is not available, please indicate this.

Not applicable.

How was the potential cultural significance of the materials collected in your study to local communities considered in your research design? Were Indigenous peoples and/or local researchers and institutions involved with archaeological excavations / collection of specimens? If so, please provide a description of their involvement.

Not applicable.

If your manuscript includes photographs of human remains please indicate whether authors obtained permission from descendants or affiliated cultural communities to do so.

Not applicable.
