# Supplementary material for: Iranian livestock breeders’ knowledge, attitude, practice, and behavioral determinants related to brucellosis prevention
Source: PLOS Glob Public Health. 2025 Oct 8;5(10):e0004693. doi: 10.1371/journal.pgph.0004693 (PMC12507205; doi:10.1371/journal.pgph.0004693)
Supplement: S1 Table — (S1_Table.DOCX) [file pgph.0004693.s003.docx]

S1 Table: The frequency (percentage) of awareness or lack of awareness of participants in response to each awareness questions

| **Awareness sub- construct** | **Item** | **Correctness of the answer** | |
| --- | --- | --- | --- |
|  |  | **No**  **(N/Percent)** | **Yes**  **(N/Percent)** |
| **Direct Awareness(AD)** | Can brucellosis be transmitted from an animal to a human? | 377 (%87.3) | 55 (%12.7) |
|  | Can brucellosis be transmitted from a sheep or a goat to a person? | 350 (%81.0) | 82 (%19.0) |
|  | Can brucellosis be transmitted from a cow to a human? | 363 (%84.0) | 69 (%16.0) |
|  | Is brucellosis transmitted from a person to another? | 341 (%78.9) | 91 (%21.1) |
|  | Can brucellosis be transmitted from skin contact with an infected animal? | 369 (%85.4) | 63 (%14.6) |
|  | Can brucellosis be transmitted through touching the aborted fetus and placenta of an infected animal? | 372 (%86.1) | 60 (%13.9) |
|  | Can wearing gloves prevent brucellosis when you contact the uterine secretions of dead animals? | 221 (%51.2) | 211 (%48.8) |
|  | Does brucellosis spread in the environment by the urine of animal? | 292 (%67.6) | 140 (%32.4) |
|  | Does brucellosis spread in the environment by the’ fetus and placenta of animal? | 374 (%86.6) | 58 (%13.4) |
|  | Does the brucellosis spread in the environment by wool? | 692 (%90.7) | 40 (%9.3) |
|  | Do you think that abortion place should be disinfected during the abortion? | 255 (%59.0) | 177 (%41.0) |
|  | Do you think a dog can eat an aborted fetus? | 334 (%77.3) | 98 (%22.7) |
|  | Do you think the aborted fetus needs to be buried? | 390 (%90.3) | 42 (%9.7) |
|  | Is brucellosis a preventable disease? | 331 (%76.6) | 101 (%23.4) |
|  | Should the place be disinfected after an abortion? | 267 (%61.8) | 165 (%38.2) |
| **Indirect Awareness(AID)** | Can brucellosis be transmitted through consuming infected milk and dairy products? | 65 (%15.0) | 367 (%85.0) |
|  | Can brucellosis be transmitted by semi-cooked meat? | 178 (%41.2) | 254 (%58.8) |
|  | Can brucellosis be transmitted through breathing into the stalls of animals? | 180 (%41.7) | 252 (%58.3) |
|  | Can washing the milking dishes prevent brucellosis? | 180 (%41.7) | 252 (%58.3) |
|  | Can boiling milk prevent brucellosis? | 213 (%49.3) | 219 (%50.7) |
|  | Can keeping cheese in salty water for two months before consumption prevent brucellosis? | 102 (%23.6) | 330 (%76.4) |
|  | Does brucellosis spread in the environment by the milk of infected livestock? | 151 (%35.0) | 281 (%65.0) |
|  | Does brucellosis spread in the environment by the meat of infected livestock? | 118 (%27.3) | 14 (%72.7) |
|  | Do you as^[[1]](#footnote-1)^k a veterinarian to help with animal abortion? | 91 (%21.1) | 341 (%78.9) |
| **Vaccine-oriented awareness(AV)** | Can livestock vaccination prevent Malta fever among humans? | 160 (%37.0) | 272 (%63.0) |
|  | Can brucellosis be prevented by vaccinating livestock? | 238 (%55.1) | 194 (%44.9) |
|  | Is animal vaccination expensive for you? | 78 (%18.1) | 354 (%81.9) |
|  | Do you have access to livestock vaccination services? | 196 (%45.4) | 236 (%54.6) |
|  | Does the veterinary organization offer timely vaccination services for your animals? | 300 (%6.4) | 132 (%30.6) |
|  | Do veterinarians encourage you to vaccinate your livestock? | 174 (%40.3) | 258 (%59.7) |
|  | Do the health personnel encourage you to vaccinate your livestock? | 250 (%57.9) | 182 (%42.1) |
|  | In which season should brucellosis vaccine be injected? | 146 (%33.8) | 286 (%66.2) |
|  | How often should the brucellosis vaccine be repeated? | 334 (%77.3) | 98 (%22.7) |
|  | At what age should livestock be vaccinated against brucellosis? | 375 (%86.8) | 57 (%13.2) |
|  | Does brucellosis affect livestock growth after vaccination? | 276 (%63.9) | 156 (%36.1) |
|  | Can livestock get brucellosis again after vaccination? | 366 (%84.7) | 66 (%15.3) |

1. Minimum= 0 , maximum= 15, sum= 1452 [↑](#footnote-ref-1)
