# Supplementary material for: Iranian livestock breeders’ knowledge, attitude, practice, and behavioral determinants related to brucellosis prevention
Source: PLOS Glob Public Health. 2025 Oct 8;5(10):e0004693. doi: 10.1371/journal.pgph.0004693 (PMC12507205; doi:10.1371/journal.pgph.0004693)
Supplement: S2 Table — (S2_Table.DOCX) [file pgph.0004693.s004.docx]

S2 Table: The number and percentage of participants who responded to each of the attitude questions

| **Construct** | **Item** | **Level of response** | | | | |
| --- | --- | --- | --- | --- | --- | --- |
|  |  | **Strongly Agree** | **Agree** | **Moderately agree** | **Disagree** | **Strongly Disagree** |
| **Attitude** | Abortion does not occur if animals are vaccinated | 12 (%2.8) | 53 (%12.3) | 19 (%4.4) | 111 (%25.7) | 237 (%54.9) |
|  | Livestock breeders should ask a veterinarian to examine their livestock. | 8 (%1.9) | 23 (%5.3) | 20 (%4.6) | 142 (%32.9) | 239 (%55.3) |
|  | I will not get brucellosis, if I touch vaccinated animals’ milk, urine, placenta and fetus. | 8 (%1.9) | 30 (%6.9) | 48 (%11.1) | 192 (%44.4) | 154 (%35.6) |
|  | As vaccination is time-consuming, I prefer getting brucellosis instead of vaccinating my animals | 14 (%3.2) | 40 (%9.3) | 61 (%14.1) | 151 (%35.0) | 166 (%38.4) |
|  | Animal vaccination is a very difficult process. | 4 (%0.9) | 52 (%12.0) | 31 (%7.2) | 122 (%28.2) | 223 (%51.6) |
|  | If I get brucellosis, I will be unable to work for a long time | 17 (%3.9) | 69 (%16.0) | 43 (%10.0) | 164 (%38.0) | 139 (%32.2) |
|  | If I do not vaccinate the animals, my family and I will get brucellosis. | 16 (%3.7) | 70 (%16.2) | 67 (%15.5) | 126 (%29.2) | 153 (%35.4) |
|  | If I vaccinate my livestock, people will not get brucellosis with consumption of my dairy products. | 8 (%1.9) | 69 (%16.0) | 34 (%7.9) | 143 (%33.1) | 178 (%41.2) |
|  | I may still be in danger of getting brucellosis, even if I do preventative measures | 33 (%7.6) | 139 (%32.2) | 55 (%12.7) | 98 (%22.7) | 107 (%24.8) |
