# Supplementary material for: Iranian livestock breeders’ knowledge, attitude, practice, and behavioral determinants related to brucellosis prevention
Source: PLOS Glob Public Health. 2025 Oct 8;5(10):e0004693. doi: 10.1371/journal.pgph.0004693 (PMC12507205; doi:10.1371/journal.pgph.0004693)
Supplement: S3 Table — (S3_Table.DOCX) [file pgph.0004693.s005.docx]

S3 Table: The number and percentage of participants’ responses to each level of practice questions

| **Construct** | **Item** | **Level of response** | | | | |
| --- | --- | --- | --- | --- | --- | --- |
|  |  | **Never** | **Rarely** | **Sometimes** | **Often** | **Always** |
| **Practice** | keep records and documents of medical history and vaccination of my livestock in a proper place | 314 (%72.7) | 46 (%10.6) | 7 (%1.6) | 27 (%6.3) | 38 (%8.8) |
|  | I have experienced abortion among my livestock | 119 (%27.5 | 179 (%41.4) | 93 (%21.5) | 17 (%3.9) | 24 (%5.6) |
|  | I always keep an eye on the cold chain about vaccines being injected into my cattle | 211 (%48.8) | 127 (%29.4) | 18 (%4.9) | 34 (%7.9) | 42 (%9.7) |
|  | I vaccinate my livestock at the proper and recommended time in a year or season | 190 (%44.0) | 140 (%32.4) | 11 (%2.5) | 63 (%14.6) | 28 (%6.5) |
|  | Every year, I vaccinate my livestock against brucellosis and I have done it for many years | 132 (%30.6) | 148 (%34.3) | 81 (%18.8) | 26 (%6.0) | 45 (%10.4) |
|  | My livestock are in contact with other non-vaccinated livestock | 122 (%28.2) | 119 (%27.5) | 68 (%15.7) | 70 (%16.2) | 53 (%12.3) |
|  | When I buy a new livestock, I am curious to know about their vaccination history | 219 (%50.7) | 118 (%27.3) | 25 (%5.8) | 34 (%7.9) | 36 (%8.3) |
|  | When I buy a new livestock, I ask a vet to examine my animal | 184 (%42.6) | 64 (%3.7) | 16 (%3.7) | 57 %13.2) | 111 (%25.7) |
