# Supplementary material for: Iranian livestock breeders’ knowledge, attitude, practice, and behavioral determinants related to brucellosis prevention
Source: PLOS Glob Public Health. 2025 Oct 8;5(10):e0004693. doi: 10.1371/journal.pgph.0004693 (PMC12507205; doi:10.1371/journal.pgph.0004693)
Supplement: Attachment — Submitted filename: Answers to the Questions.docx [file pgph.0004693.s008.docx]

Answers to the Questions:

1. Provide a clear explanation for why the sample consisted exclusively of male respondents. Also address how this limitation may affect the findings and consider discussing the potential impact on the generalizability of the results.

Answer: The present staudy has some limitations. Firstly, In Iran, livestock breeding remains a male-dominated sector, particularly in rural villages, reflecting deep-rooted cultural, social, and religious norms. This gender disparity is further reinforced by the physically demanding nature of livestock management, which involves handling animals, transporting heavy feed, and performing labor-intensive tasks over extended hours in often harsh outdoor conditions.These demanding conditions align with the prevailing societal belief that physically heavy work is more appropriate for men. Only in some border regions, where nomadic communities migrate seasonally, do women participate in activities such as milking and feeding livestock. However, as previously noted, livestock breeding in Iran is predominantly male-dominated. At our study site, all livestock breeders were male. Therefore, the findings of this study can be generalized within the Iranian context, but may not be applicable to other countries.
