# Supplementary material for: Iranian livestock breeders’ knowledge, attitude, practice, and behavioral determinants related to brucellosis prevention
Source: PLOS Glob Public Health. 2025 Oct 8;5(10):e0004693. doi: 10.1371/journal.pgph.0004693 (PMC12507205; doi:10.1371/journal.pgph.0004693)
Supplement: Attachment — Submitted filename: Response to Reviewers.docx [file pgph.0004693.s010.docx]

**Response to Reviewers:**

**6. Review Comments to the Author**

**Reviewer #2: It was a pleasure for me to be one of the reviewers of this manuscript. I have read it extensively and several times, and I can recommend it to everyone I know. The manuscript meets the PLOS GLOBAL PUBLIC HEALTH CRITERIA exactly. I fully understand the conclusion of the manuscript, as it is based on the data presented. I would like to see and read studies of this kind more often. It is very important that people understand the causes, modes of transmission and prevention of brucellosis, as it is a disease that poses a risk to public health.**

**Answer**: Thank you very much for your thoughtful and generous feedback. We are truly honored by your kind words and deeply appreciate the time and effort you devoted to reviewing our manuscript. Your recognition of the study's importance and relevance to public health, especially regarding the prevention and understanding of brucellosis, is greatly encouraging. We hope that this work will contribute meaningfully to both scientific understanding and public awareness of this significant health issue. Thank you once again for your valuable support.

**Reviewer #3: Brucellosis is one of the most important public health challenges in low- and middle-income countries.The article is of good quality in terms of subject matter, method, and analysis, and can contribute to the field of livestock health and brucellosis control. However, some methodological and writing points require minor correction:**

**Answer:** Thank you very much for your constructive and encouraging feedback. We truly appreciate your acknowledgment of the relevance and quality of our study, particularly its potential contribution to livestock health and brucellosis control in low- and middle-income countries. We also value your observations regarding methodological and writing aspects that require minor corrections. In response, we have carefully reviewed and revised the manuscript to address these points and improve overall clarity and precision. Thank you once again for your valuable input and support.

1- **The article should better state its innovation more clearly in the introduction compared to previous studies in Iran.**

**Answer:** Thank you for your comment. I hope I have interpreted it correctly and made the necessary revision. The change has been implemented in line 111 on page 6.

**2-Due to the use of interviews with illiterate individuals, there is a possibility of interviewer bias; this should be highlighted in the limitations section. It would be better to provide more information on how to train interviewers and control data entry errors.**

**Answer: Thank you for your insightful comment. We agree that the use of interviews with illiterate participants could introduce interviewer bias. We have now acknowledged this as a potential limitation in the revised manuscript (see Limitations section). We also implemented double data entry procedures and random checks to minimize errors during data processing.**

**3-****It would have been better to use multivariate regression analysis for all demographic variables to examine the adjusted effects more precisely.**

**Answer:** **Thank you for your valuable comment.**
In this study, our primary aim in conducting the KAP (Knowledge, Attitudes, and Practices) analysis was not to predict outcomes or establish causal relationships between demographic variables and KAP components, but rather to provide a descriptive overview of the current situation in the study area. Therefore, we did not apply multivariate regression analysis in this particular paper.

However, we acknowledge the importance of adjusted analyses for more precise estimations. In other publications derived from the same dataset, we have employed regression methods to explore such relationships in greater depth.

**4-Practical suggestions for policymakers and veterinary institutions could be clearer.**

**Answer:** Thank you for your valuable comment. We have now revised the implication section to include clearer and more specific practical recommendations for policymakers and veterinary institutions.Line 393 page 18.

**5-Some parts of the discussion are very long and should be structured in a more concise and targeted manner.**

**Answer:** Thank you for your insightful feedback. In response, we have revised the Discussion section to improve clarity and focus. We have removed repetitive content, streamlined overly long paragraphs, and structured the section in a more concise and targeted manner to better highlight the key findings and their implications.

**6-Need for minor spelling revision in some English sentences (e.g. using correct tenses in verbs)**

Answer: Thank you for your observation. We appreciate your attention to language accuracy. We have carefully reviewed the manuscript and revised the text to correct verb tenses and minor spelling errors to ensure clarity and proper academic tone.
